# Supplementary material for: Comparative effectiveness of nonpharmacological interventions for the nutritional status of maintenance hemodialysis patients: a systematic review and network meta-analysis
Source: PeerJ. 2025 Feb 24;13:e19053. doi: 10.7717/peerj.19053 (PMC11867038; doi:10.7717/peerj.19053)
Supplement: Supplemental Information 3 [file peerj-13-19053-s003.docx]

**Supplementary Online Content**

**Contents**

Appendix A. Search String 3

Appendix B. Reference list of included studies 5

Appendix C. Bayesian MCMC chain trajectory map and density map 10

1 BMI 10

2 ALB 11

2 HB 12

Appendix D. Iterative convergence Brooks - Gelman - Rubin diagnostic graph 12

1 BMI 12

2 ALB 13

2 HB 13

Appendix E. Assessment of Inconsistency 13

Appendix F. Assessment of Model fit 14

Appendix G. Results of the network rank test (SUCRA) 15

Appendix H. Probability plot ranking of different interventions on outcome indicators 16

1 BMI 16

2 ALB 17

3 HB 18

Appendix I. Comparison-adjusted funnel plots 19

1 BMI 19

2 ALB 20

3 HB 21

Appendix J. Sensitivity Analyses for Outcomes 22

Appendix K. GRADE Ratings for All Contrasts 24

1 BMI 24

2 ALB 24

3 HB 25

***Appendix A. Search String***

Take PubMed as an example. The following keywords and Medical Subject Headings (MESH) will be used for searching articles:

| #1 | (((((((((((((((Renal Dialysis[MeSH Terms]) OR (Hemodialysis[Title/Abstract])) OR (Hemodialyses[Title/Abstract])) OR (Extracorporeal Dialyses[Title/Abstract])) OR (Extracorporeal Dialysis[Title/Abstract])) OR (maintenance hemodialysis[Title/Abstract])) OR (Dialysis[MeSH Terms])) OR (Dialyses[Title/Abstract])) OR (Hemoperfusion[MeSH Terms])) OR (Hemosorption[Title/Abstract])) OR (Kidney Failure, Chronic[MeSH Terms])) OR (End-Stage Kidney Disease[Title/Abstract])) OR (End-Stage Renal Disease[Title/Abstract])) OR (End-Stage Renal Failure[Title/Abstract])) OR (Renal Failure, Chronic[Title/Abstract])) OR (ESRD[Title/Abstract]) |
| --- | --- |
| #2 | ((((((((((((((((((Exercise[MeSH Terms]) OR (Physical Activity[Title/Abstract])) OR (Physical Activities[Title/Abstract])) OR (Physical Exercise[Title/Abstract])) OR (Acute Exercise[Title/Abstract])) OR (Isometric Exercises[Title/Abstract])) OR (Aerobic Exercise[Title/Abstract])) OR (Exercise Trainings[Title/Abstract])) OR (Psychology[MeSH Terms])) OR (Psychological Side Effect[Title/Abstract])) OR (Psychosocial Factor[Title/Abstract])) OR (Psychologist[Title/Abstract])) OR (diet[MeSH Terms])) OR (diets[Title/Abstract])) OR (dietary[Title/Abstract])) OR (Health Education[MeSH Terms])) OR (Community Health Education[Title/Abstract])) OR (oral nutritional supplement[Title/Abstract])) OR (ONS[Title/Abstract]) |
| #3 | (((((((Nutritional Status[MeSH Terms]) ) OR (Nutrition Status[Title/Abstract])) OR (Malnutrition[MeSH Terms])) OR (Nutritional Deficiency[Title/Abstract])) OR (Nutritional Deficiencies[Title/Abstract])) OR (Undernutrition[Title/Abstract])) OR (Malnourishment[Title/Abstract]) |
| #4 | #1 AND #2 AND #3 |

Take CNKI as an example, the following keywords and Medical Subject Headings (MESH) will be used for searching articles:

SU: maintenance hemodialysis OR hemodialysis OR renal dialysis OR blood dialysis OR Hemoperfusion OR End-stage renal disease

SU: exercise OR psychology OR diet OR health education OR oral nutritional supplements Search in results

SU: nutrition OR nutritional status OR malnutrition OR nutritional deficiency retrieved Search in results

***Appendix B.*** ***Reference list of included studies***

1. Wei 2020

- Wei, Q., 2020. Observation on the effect of exercise therapy with nursing intervention on the nutritional status and quality of life of patients on maintenance hemodialysis. World Latest Medicine Information 20 (7), 297-298.

1. Zhou et al. 2016

- Zhou, R., Zhou, P., Li, Q., Zheng, J., Li, Y., Li, J., Wen, N., Liao, L., 2016. Effect of nursing intervention combined with exercise therapy on the quality of life of hemodialysis patients. Guangxi Medical Journal 38 (01), 134-136+139.

1. Li et al. 2008

- Li, J., Tu, E., Guo, Y., Liu, P., 2008. Application of nutrition guidance in maintenance of hemodialysis patients Chinese Journal of Modern Nursing 14 (11), 1248-1250.

1. Lu 2022

- Lu, M., 2022. A study on the nutritional status of MHD patients with traditional Chinese medicine behavioral intervention and health management. Liaoning University of Traditional Chinese Medicine.

1. Abreu et al. 2017

- Abreu, C.C., Cardozo, L., Stockler-Pinto, M.B., Esgalhado, M., Barboza, J.E., Frauches, R., Mafra, D., 2017. Does resistance exercise performed during dialysis modulate Nrf2 and NF-κB in patients with chronic kidney disease? Life Sci 188, 192-197.

1. Wang et al. 2019

- Wang, X., Zhang, K., Ge, Y., Wang, A., Yu, C., 2019. Effects of intradialytic exercise on inflammation, oxidative stress and endothelial function in patients with maintenance hemodialysis Chinese Journal of Blood Purification 18 (6), 390-393.

1. Afshar et al. 2010

- Afshar, R., Shegarfy, L., Shavandi, N., Sanavi, S., 2010. Effects of aerobic exercise and resistance training on lipid profiles and inflammation status in patients on maintenance hemodialysis. Indian J Nephrol 20 (4), 185-189.

1. Dai et al 2022

- Dai, S., Ma, Y., 2021. Effect of intradialytic progressive resistance training on nutritional status and body composition in maintenance hemodialysis patients. Chinese Journal of Nephrology 37 (5), 434-437.

1. Yan et al. 2022
   - Yan, X., Zhao, Q., Peng, Y., 2022. Effects of step-by-step resistance exercise on exercise capacity, nutritional indexes, and sleep quality in maintenance hemodialysis patients. Chinese Evidence-based Nursing 8 (9), 1215-121
2. Cai et al 2022
   - Cai, G., Hu, J., Wang, Q., Gao, Y., Feng, C., Wu, W., Chang, L., Chai, C., 2022. Effect of exercise rehabilitation on nutritional status and fatigue syndrome in maintenance hemodialysis patients. China Modern Medicine 29 (16), 140-143+147.
3. Tayebi et al. 2018

- Tayebi, M., Ramezani, A., Kashef, M., 2018. The Effect of Intradialytic Isometric Resistance Training on Muscle Capacity and Serum Albumin Levels in Hemodialysis Patients. Nephro-Urology Monthly In Press (In Press).

1. Liao et al. 2016
   - Liao, M.T., Liu, W.C., Lin, F.H., Huang, C.F., Chen, S.Y., Liu, C.C., Lin, S.H., Lu, K.C., Wu, C.C., 2016. Intradialytic aerobic cycling exercise alleviates inflammation and improves endothelial progenitor cell count and bone density in hemodialysis patients. Medicine (Baltimore) 95 (27), e4134.
2. Leng 2012
   - Leng, C., 2012. The effect of aerobic exercise on the intervention of maintenance hemodialysis patients.
3. Wang et al. 2021
   - Wang, C., Liu, Y., 2021. Effects of Aerobic Exercise on Nutritional Status, Psychological Status and Quality of Life in Patients with Maintenance Hemodialysis. World Latest Medicine Information 21 (105), 274-276.
4. Wilund et al. 2010
   - Wilund, K.R., Tomayko, E.J., Wu, P.T., Ryong Chung, H., Vallurupalli, S., Lakshminarayanan, B., Fernhall, B., 2010. Intradialytic exercise training reduces oxidative stress and epicardial fat: a pilot study. Nephrol Dial Transplant 25 (8), 2695-27
5. Su et al. 2022
   - Su, X., Ai, L., Duan, Y., Song, C., Li, W., 2022. Effect of Aerobic Exercise on Resting Energy Metabolism, PEW Status and Cardiopulmonary Endurance in Patients with Maintenance Hemodialysis. Chinese Journal of Dialysis and Artificial Organs 33 (02), 96-101.
6. Wang et al. 2023
   - Wang, Y., Zhao, Q., Luo, Y., Feng, X., Zhang, F., Yu, X., Liu, F., Xu, J., Wu, G., Chen, Q., 2023. Clinical study on the improvement of protein depletion in hemodialysis patients by the intervention of oral nutritional supplement. Chinese Journal of Blood Purification 22 (01), 11-16.
7. Chen et al. 2019
   - Chen, R., Zhao, X., Huang, X., 2019. Survey on nutritional status and application of oral nutritional supplement among maintenance hemodialysis patients. Chinese Journal of Practical Nursing 35 (13), 980-985.
8. Jeong et al. 2019
   - Jeong, J.H., Biruete, A., Tomayko, E.J., Wu, P.T., Fitschen, P., Chung, H.R., Ali, M., McAuley, E., Fernhall, B., Phillips, S.A., Wilund, K.R., 2019. Results from the randomized controlled IHOPE trial suggest no effects of oral protein supplementation and exercise training on physical function in hemodialysis patients. Kidney Int 96 (3), 777-786.
9. Limwannata et al 2021
   - Limwannata, P., Satirapoj, B., Chotsriluecha, S., Thimachai, P., Supasyndh, O., 2021. Effectiveness of renal-specific oral nutritional supplements compared with diet counseling in malnourished hemodialysis patients. Int Urol Nephrol 53 (8), 1675-1687.
10. Bolasco et al. 2011
    - Bolasco, P., Caria, S., Cupisti, A., Secci, R., Saverio Dioguardi, F., 2011. A novel amino acids oral supplementation in hemodialysis patients: a pilot study. Ren Fail 33 (1), 1-5.
11. Tan et al. 2015
    - Tan, R., Ji, L., Zhong, X., Qin, D., He, Y., Zeng, T., He, H., Liu, Y., 2015. Effect of oral nutrition supplement on nutritional status in maintenance hemodialysis patients with protein-energy wasting Chinese Journal of Blood Purification 14 (09), 550-553.
12. Yang et al 2021
    - Yang, Y., Qin, X., Chen, J., Wang, Q., Kong, Y., Wan, Q., Tao, H., Liu, A., Li, Y., Lin, Z., Huang, Y., He, Y., Lei, Z., Liang, M., 2021. The Effects of Oral Energy-Dense Supplements on Nutritional Status in Nondiabetic Maintenance Hemodialysis Patients: A Randomized Controlled Trial. Clin J Am Soc Nephrol 16 (8), 1228-1236.
13. Wen et al. 2022
    - Wen, L., Tang, C., Liu, Y., Jiang, J., Zou, D., Chen, W., Xu, S., Wang, Y., Qiu, J., Zhong, X., Liu, Y., Tan, R., 2022. Effects of oral non-protein calorie supplements on nutritional status among maintenance hemodialysis patients with protein-energy wasting: a multi-center randomized controlled trial. Food Funct 13 (16), 8465-8473.
14. Sezer et al. 2014
    - Sezer, S., Bal, Z., Tutal, E., Uyar, M.E., Acar, N.O., 2014. Long-term oral nutrition supplementation improves outcomes in malnourished patients with chronic kidney disease on hemodialysis. JPEN J Parenter Enteral Nutr 38 (8), 960-965.
15. Yu et al. 2018
    - Yu, J., Cao, Z., 2018. Effect of oral nutritional supplements on improving nutritional status and clinical outcomes of patients with hemodialysis. Journal of Clinical Medicine in Practice 22 (18), 25-27
16. Ai 2020
    - Ai, X., 2020. Effects of individualized dietary care on nutritional status and immune function of maintenance hemodialysis patients. Modern Diagnosis & Treatment 31 (05), 807-808.
17. Sun et al. 2022
    - Sun, M., Sun, X., Yang, L., 2022. Precise diet management for patients undergoing maintenance hemodialysis. Journal of Nursing Science 37 (10), 20-22.
18. Zhou 2020
    - Zhou, F., 2020. Observation on the effect of dietary care intervention on nutritional status and quality of life of maintenance hemodialysis patients. Electronic Journal of Practical Clinical Nursing Science 5 (30), 152,165.
19. Xu et al. 2022
    - Xu, X., Huang, C., Liang, X., Li, L., 2022. To study the effect of personalized dietary intervention on maintenance hemodialysis patients. Psychologies Magazine 17 (18), 180-182+194.
20. Wang 2019
    - Wang, Y., 2019. Effects of dietary nursing intervention on nutritional status, quality of life and adherence of maintenance hemodialysis patients. The Journal of Medical Theory and Practice 32 (03), 451-453.
21. Wang 2018
    - Wang, L., 2018. Effect of dietary intervention on calcium and phosphorus metabolism and nutritional status of elderly maintenance hemodialysis patients. Journal of Tanshan Medical College 39 (03), 308-309.
22. Deng 2011
    - Deng, Z., 2011. The effect of dietary intervention on the quality of survival of maintenance hemodialysis patients. International Journal of Nursing Studies 30 (2), 188-189.
23. Li et al. 2020
    - Li, F., Feng, Z., 2020. Exploring the effect of personalized dietary intervention on urea clearance index in uremic hemodialysis patients. Modern Nurse 27 (4), 41-43.
24. Xu et al. 2016
    - Xu, Y., Fang, Y., 2016. Effect of diet intervention on nutritional status of elderly patients on maintenance hemodialysis. Hainan Medical Journal 27 (05), 858-860.
25. Kozlowska et al. 2023
    - Kozlowska, L., Jagiello, K., Ciura, K., Sosnowska, A., Zwiech, R., Zbrog, Z., Wasowicz, W., Gromadzinska, J., 2023. The Effects of Two Kinds of Dietary Interventions on Serum Metabolic Profiles in Haemodialysis Patients. Biomolecules 13 (5).
26. Feng et al. 2020
    - Feng, L., Yang, J., Li, P., Li, Y., Chen, K., 2020. Study on the improvement of malnutrition and mineral bone metabolism in dialysis patients by electronic intemgent assistant tools Chinese Journal of Blood Purification 19 (05), 301-304.
27. Zeng et al. 2020
    - Zeng, T., Zhang, N., Yang, L., Ding, W., 2020. Influence of personalized nutrition management based on network platform on the patients with hemodialysis malnutrition. China Medical Herald 17 (02), 160-164.
28. Shi et al. 2021
    - Shi, S., Wang, P., Zhou, Q., You, L., Li, Y., Lin, Q., Wang, J., 2021. Application of “Internet +” nutrition education on patients with maintenance hemodialysis. Chinese Journal of Nursing 56 (01), 33-39.
29. Vijaya et al. 2019
    - Vijaya, K.L., Aruna, M., Narayana Rao, S.V.L., Mohan, P.R., 2019. Dietary Counseling by Renal Dietician Improves the Nutritional Status of Hemodialysis Patients. Indian J Nephrol 29 (3), 179-185.
30. Yao et al. 2020
    - Yao, J., Xu, L., Wu, C., Wu, J., You, G., 2020. Effects of dietary health education on dietary management behavior, calcium and phosphorus metabolism and nutritional status of maintenance hemodialysis patients. Chinese Journal of Health Education 36 (12), 1141-1144.
31. Fakhrpour et al. 2020
    - Fakhrpour, R., Hamid Tayebi Khosroshahi, H., Ebrahim, K., Ahmadizad, S., Abbasnejad, M., Mesgari Abbasi, M., Ghanbari, A., Yaghoobi, S.F., 2020. Effect of Sixteen Weeks Combined Training on FGF-23, Klotho, and Fetuin-A Levels in Patients on Maintenance Hemodialysis. Iran J Kidney Dis 14 (3), 212-218.
32. Tabibi et al. 2023
    - Tabibi, M.A., Cheema, B., Salimian, N., Corrêa, H.L., Ahmadi, S., 2023. The effect of intradialytic exercise on dialysis patient survival: a randomized controlled trial. BMC Nephrol 24 (1), 100.
33. Fang et al. 2023
    - Fang, M., Dai, M., Yang, W., Zhang, C., Sun, C., 2023. Effect of aerobic combined with resistance exercise on elderly maintenance hemodialysis patients with sarcopenic obesity Journal of Nursing Science 38 (05), 95-100.
34. Frith et al 2017
    - Frih, B., Jaafar, H., Mkacher, W., Ben Salah, Z., Hammami, M., Frih, A., 2017. The effect of interdialytic combined resistance and aerobic exercise training on health-related outcomes in chronic hemodialysis patients: the Tunisian randomized controlled study. Frontiers in Physiology 8, 288.
35. Zhu et al. 2020
    - Zhu, L., Lu, M., Wang, H., Zhao, D., Ma, R., Wang, X., 2020. Effects of planned aerobic-resistance exercise on patients' nutritional status and intra-dialytic hypotension during dialysis. Chinese Journal of Modern Nursing 26 (14), 1894-1898.
36. Martin-Alemañy et al 2020
    - Martin-Alemañy, G., Espinosa-Cuevas, M., Pérez-Navarro, M., Wilund, K.R., Miranda-Alatriste, P., Cortés-Pérez, M., García-Villalobos, G., Gómez-Guerrero, I., Cantú-Quintanilla, G., Ramírez-Mendoza, M., Valdez-Ortiz, R., 2020. Effect of Oral Nutritional Supplementation With and Without Exercise on Nutritional Status and Physical Function of Adult Hemodialysis Patients: A Parallel Controlled Clinical Trial (AVANTE-HEMO Study). J Ren Nutr 30 (2), 126-136.
37. Martin-Alemañy et al 2016
    - Cantu-Quintanilla, G., Lopez-Alvarenga, J.C., Miranda-Alatriste, P., Espinosa-Cuevas, A., 2016. The effects of resistance exercise and oral nutritional supplementation during hemodialysis on indicators of nutritional status and quality of life. Nephrol Dial Transplant 31 (10), 1712-1720.
38. Hristea et al. 2016
    - Hristea, D., Deschamps, T., Paris, A., Lefrançois, G., Collet, V., Savoiu, C., Ozenne, S., Coupel, S., Testa, A., Magnard, J., 2016. Combining intra-dialytic exercise and nutritional supplementation in malnourished older hemodialysis patients: Towards better quality of life and autonomy. Nephrology (Carlton) 21 (9), 785-790.
39. Martin-Alemañy et al 2022
    - Martin-Alemañy, G., Perez-Navarro, M., Wilund, K.R., García-Villalobos, G., Gómez-Guerrero, I., Cantú-Quintanilla, G., Reyes-Caldelas, M.A., Espinosa-Cuevas, A., Escobedo, G., Medeiros, M., Bennett, P.N., Valdez-Ortiz, R., 2022. Effect of Intradialytic Oral Nutritional Supplementation with or without Exercise Improves Muscle Mass Quality and Physical Function in Hemodialysis Patients: A Pilot Study. Nutrients 14 (14).
40. Dong et al. 2011
    - Dong, J., Sundell, M.B., Pupim, L.B., Wu, P., Shintani, A., Ikizler, T.A., 2011. The effect of resistance exercise to augment long-term benefits of intradialytic oral nutritional supplementation in chronic hemodialysis patients. J Ren Nutr 21 (2), 149-159.

# ***Appendix C. Bayesian MCMC chain trajectory map and density map***

## *1 BMI*


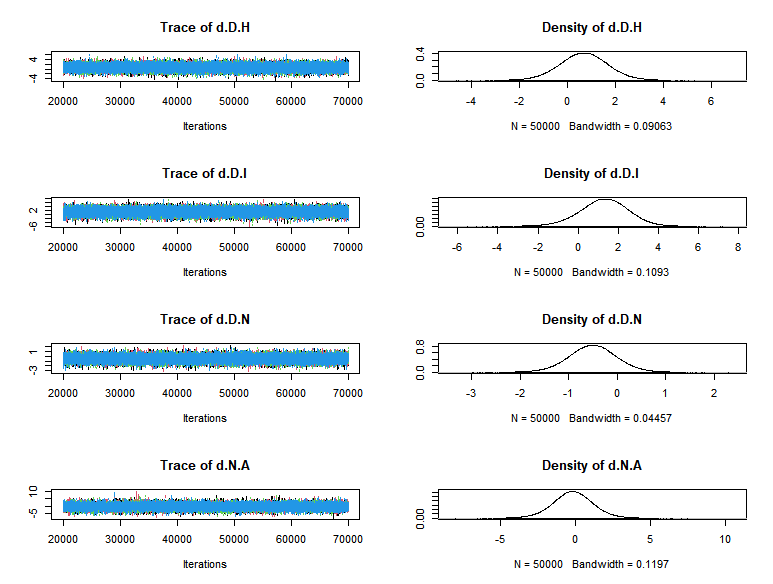

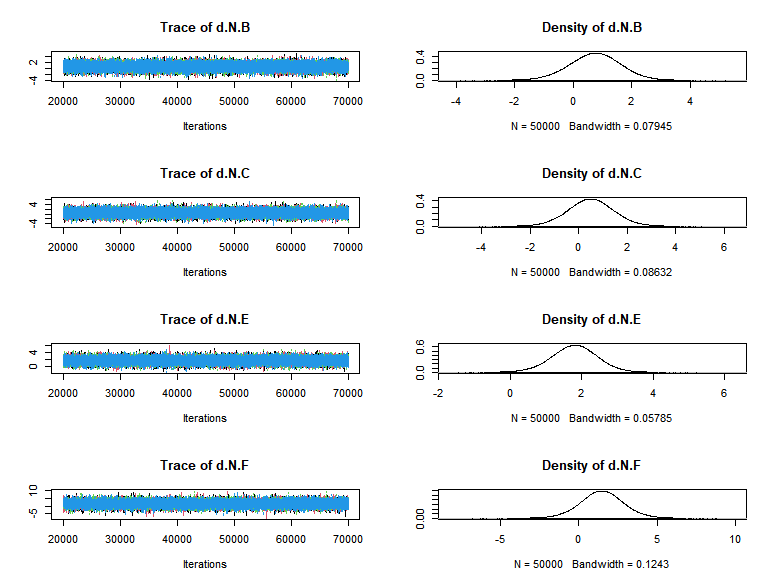

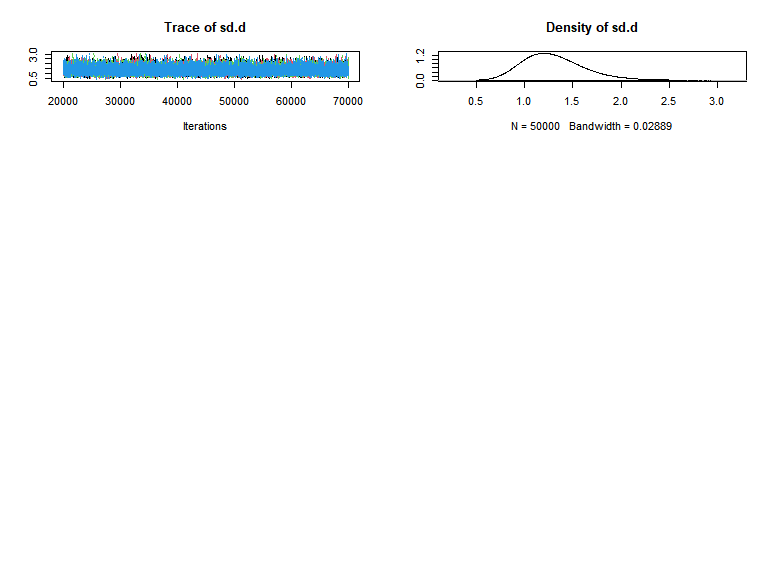


## *2 ALB*


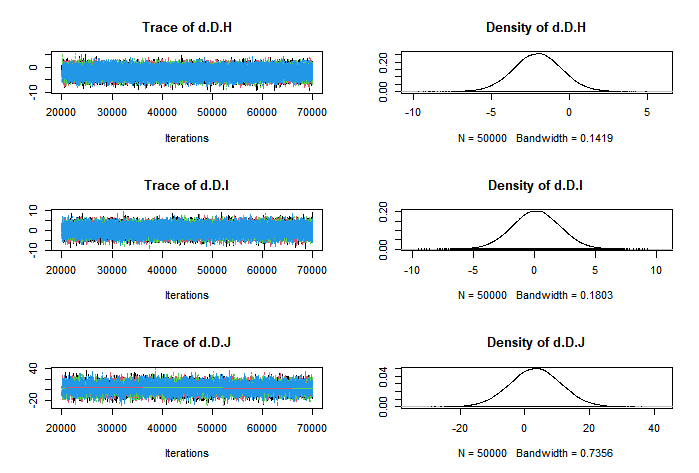

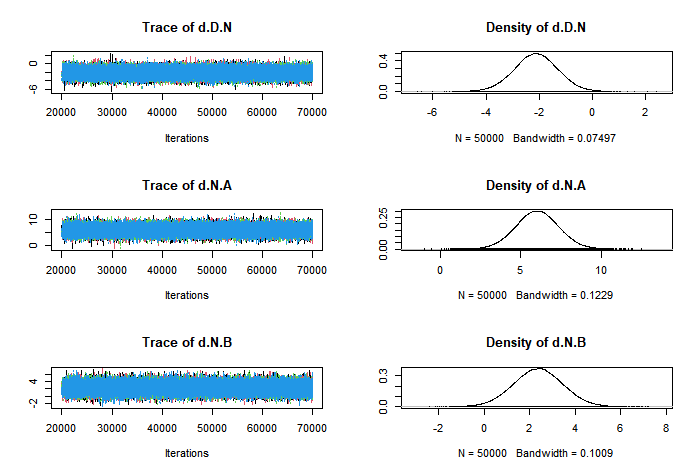

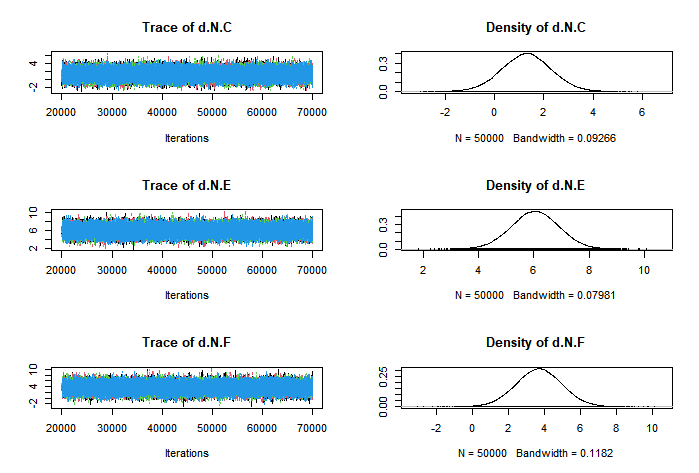

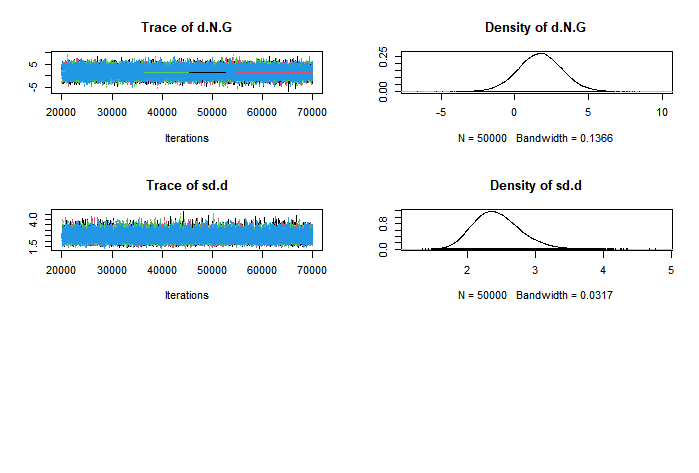


## *3 HB*


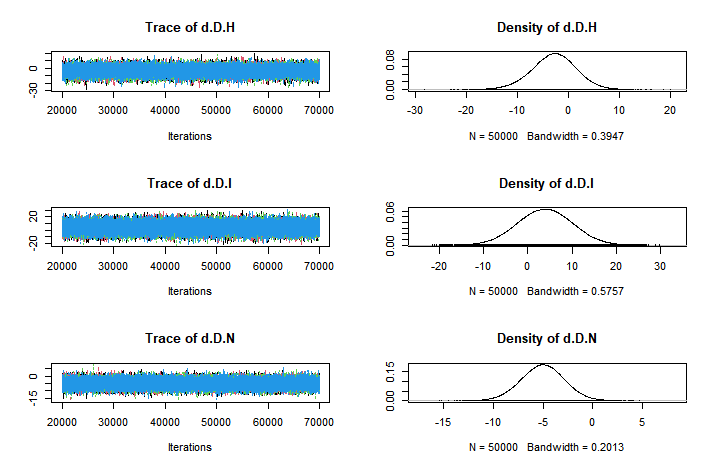

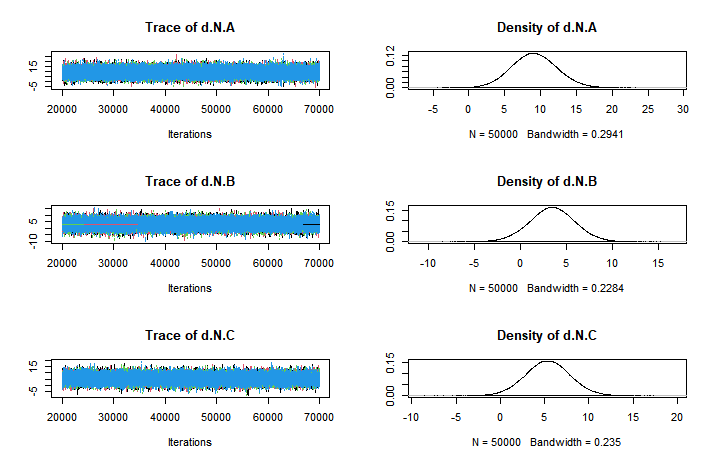

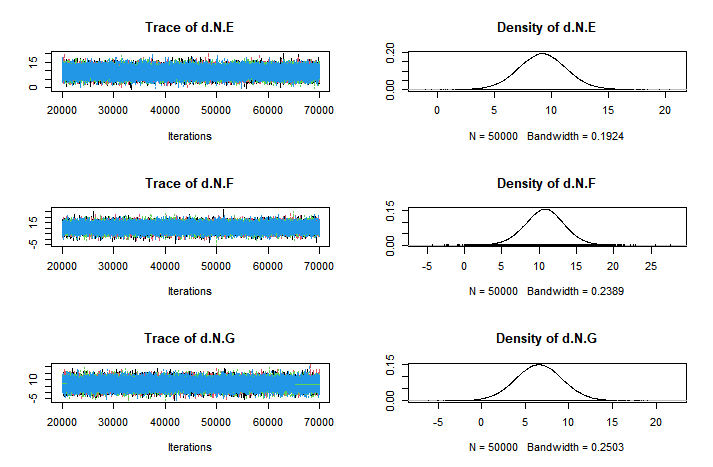

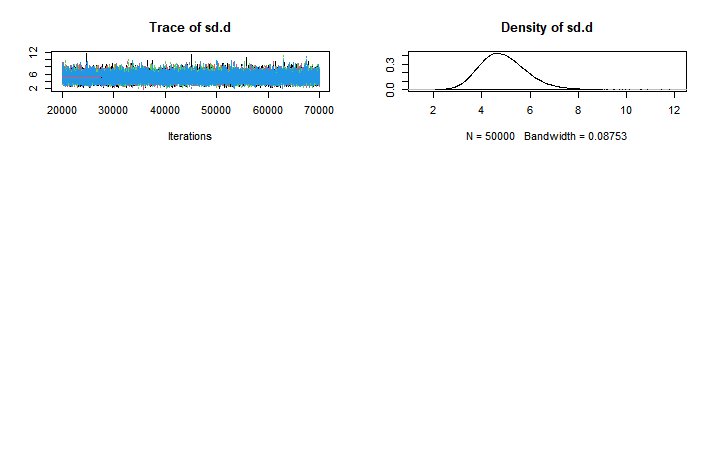


# ***Appendix D. Iterative convergence Brooks - Gelman - Rubin diagnostic graph***

## *1 BMI*


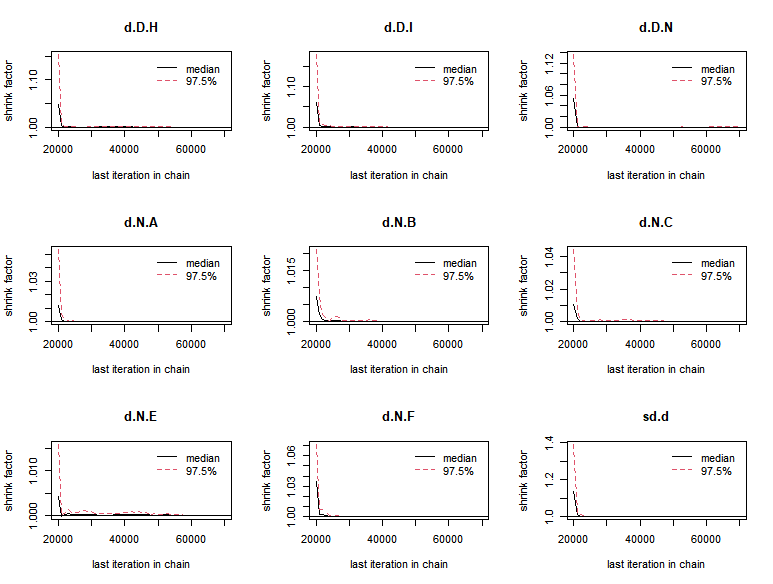


## *2. ALB*


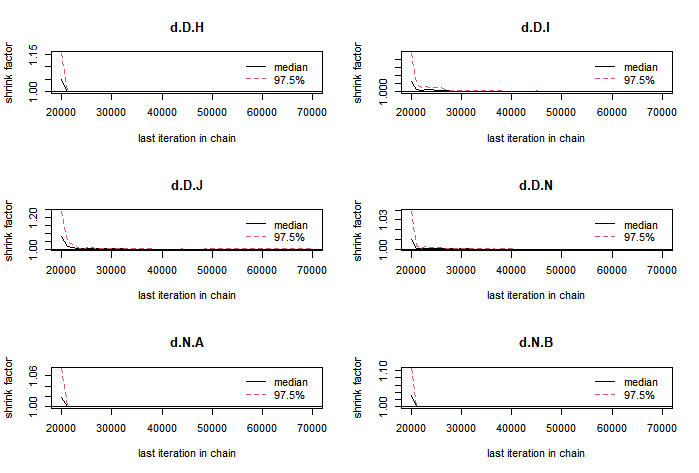

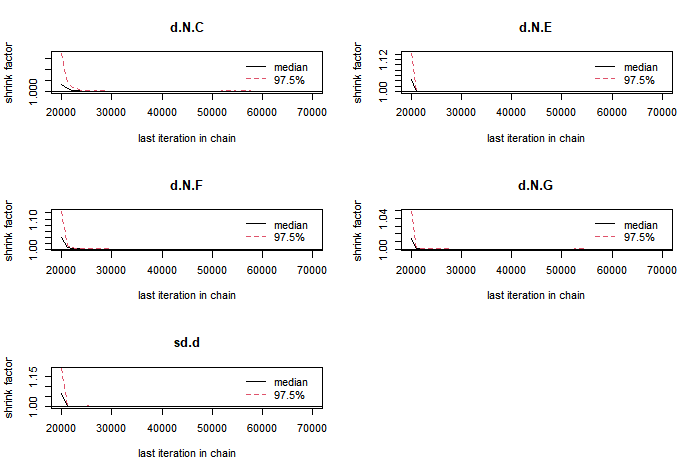


## *3 HB*


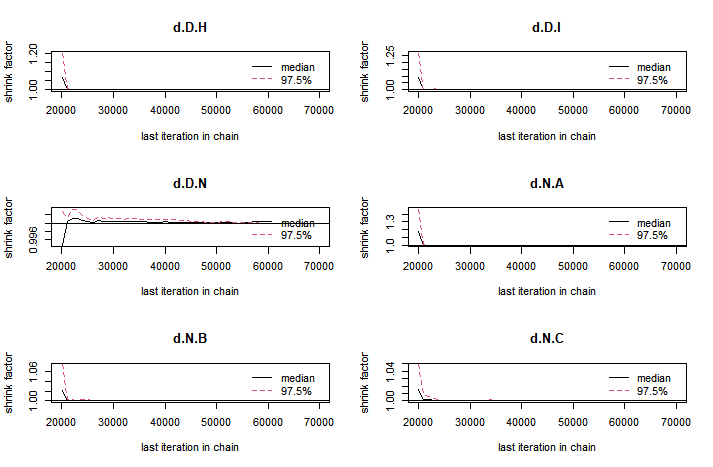

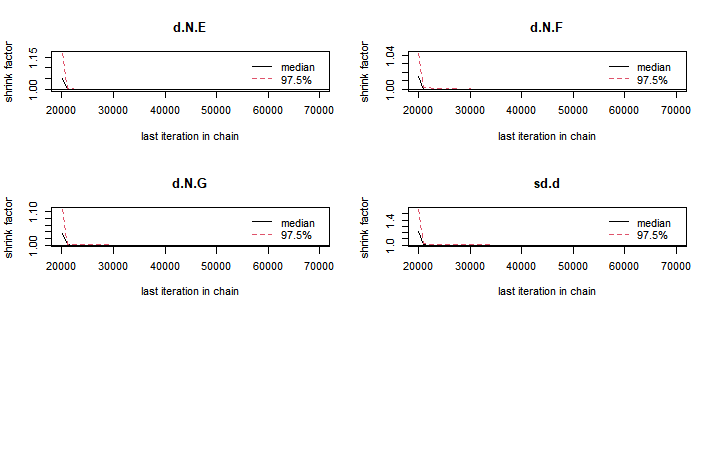


# ***Appendix E. Assessment of Inconsistency***

## *1 BMI*

| comparison | p.value | CrI |
| --- | --- | --- |
| d. H. I | 0.22 | -- |
| direct | -- | 1.1 (-2.0, 4.2) |
| indirect | -- | -3.1 (-9.1, 2.9) |
| network | -- | 0.66 (-2.1, 3.2) |
| d. H. N | 0.79 |  |
| direct | -- | -1.6 (-6.4, 3.2) |
| indirect | -- | -0.88 (-3.4, 1.7) |
| network | -- | -1.2 (-3.3, 1.0) |

## *2 ALB*

| comparison | p.value | CrI |
| --- | --- | --- |
| d. B. C | 0.68 | -- |
| direct | -- | 0.0046(-6.2, 6.3) |
| indirect | -- | -1.4 (-4.8, 1.8) |
| network | -- | -1.1 (-3.9, 1.7) |
| d. H. N | 0.80 | -- |
| direct | -- | 0.72(-4.5, 5.9) |
| indirect | -- | -0.14(-4.6, 4.4) |
| network | -- | -0.073 (-3.4, 3.2) |
| d. H. I | 0.51 | -- |
| direct | -- | 4.0(-2.5, 11) |
| indirect | -- | 1.0(-5.5, 7.5) |
| network | -- | 2.2(-2.3, 6.7) |

## *3 HB*

| comparison | p.value | CrI |
| --- | --- | --- |
| d. B. C | 0.35 | -- |
| direct | -- | -2 (-13, 8.8) |
| indirect | -- | 4.2 (-4.1, 13) |
| network | -- | 1.9 (-4.8, 8.5) |
| d. H. I | 0.07 | -- |
| direct | -- | 19(-.057, 39) |
| indirect | -- | -7.8(-29, 13) |
| network | -- | 6.8(-7.1, 21) |

Notes: B = Resistance exercise; C = Aerobic exercise; H= Oral nutritional supplementation combined with Aerobic exercise; I= Oral nutritional supplementation combined with Resistance exercise; N = Usual care.

# ***Appendix F. Assessment of Model fit***

PD: It refers to the effective number of parameters, which can show the complexity of the model.

DIC: deviance information criterion， DIC

| Outcomes | Model | pD | DIC | I^2^ |
| --- | --- | --- | --- | --- |
| BMI | **Random effects** | 50.22 | 108.79 | 3% |
|  | Fixed effects | 36.03 | 149.94 | 50% |
| ALB | **Random effects** | 90.14 | 188.19 | 2% |
|  | Fixed effects | 57.03 | 373.38 | 70% |
| HB | **Random effects** | 72.54 | 158.22 | 3% |
|  | Fixed effects | 50.04 | 221.35 | 52% |

# ***Appendix G. Results of the network rank test*** ***(SUCRA)***

| Treatment | BMI (%) | ALB (%) | HB (%) |
| --- | --- | --- | --- |
| Comprehensive nursing care | 25.34 % | 88.00 % | 72.87% |
| Resistance exercise | 47.58 % | 49.62 % | 29.43% |
| Aerobic exercise | 39.87 % | 32.39% | 44.59% |
| ONS | 39.13 % | 46.33% | 41.68% |
| Dietary intervention | 79.25 % | 89.10% | 75.49% |
| Health education | 65.42 % | 66.74% | 84.40% |
| Aerobic combined with Resistance exercise | -- | 39.17% | 54.21% |
| ONS combined with Aerobic exercise | 59.45% | 17.08% | 24.98% |
| ONS combined with Resistance exercise | 74.49% | 46.88 % | 66.38% |
| ONS combined with Aerobic and Resistance exercise | -- | 63.57% | -- |
| Usual care | 19.47% | 11.10% | 5.98% |

Notes: ONS= Oral nutritional supplementation

# ***Appendix H. Probability plot ranking of different interventions on outcome indicators***

## *1 BMI*


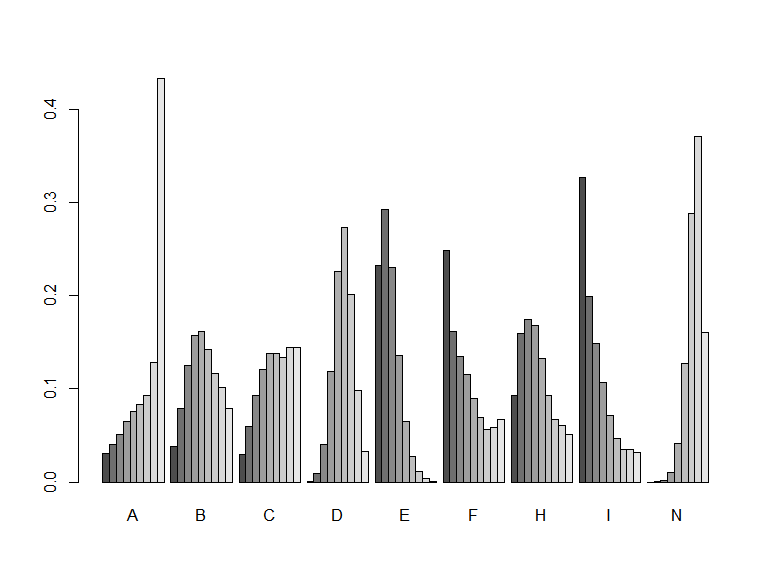

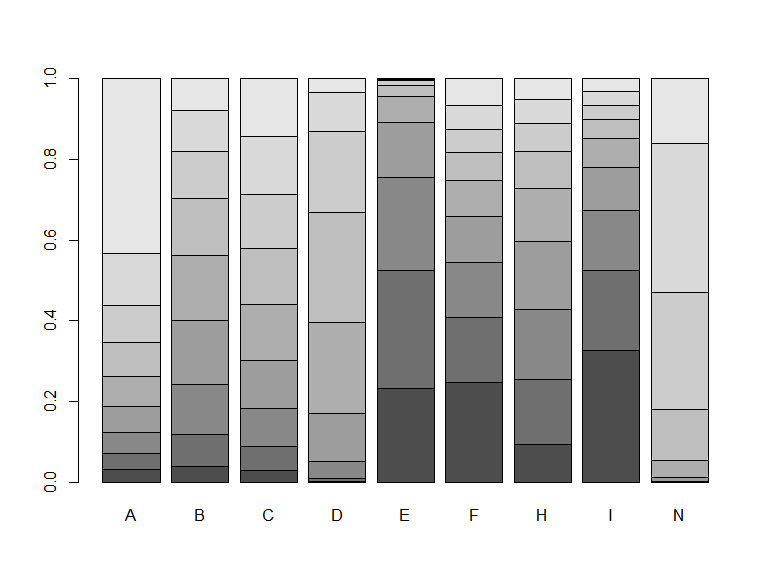


Notes: A= Comprehensive nursing care; B = Resistance exercise; C = Aerobic exercise; D= Oral nutritional supplementation; E = Dietary intervention; F=Health education; H= Oral nutritional supplementation combined with Aerobic exercise; I= Oral nutritional supplementation combined with Resistance exercise; N= Usual care.

## *2 ALB*


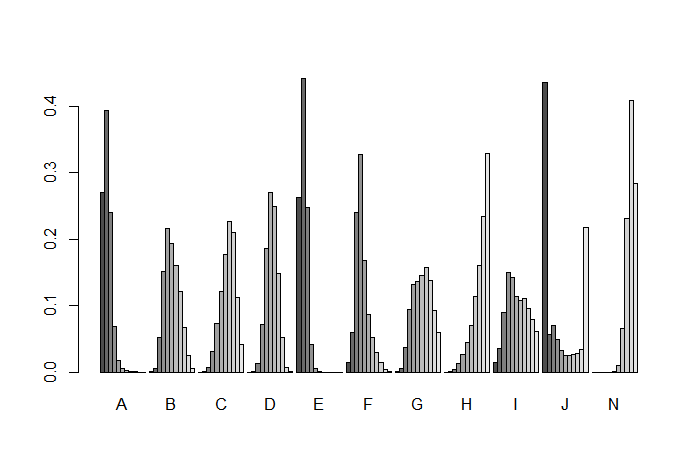

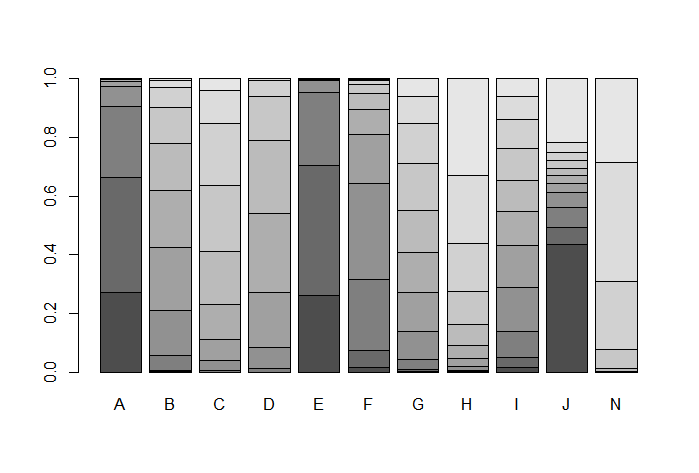


Notes: A= Comprehensive nursing care; B = Resistance exercise; C = Aerobic exercise; D= Oral nutritional supplementation; E = Dietary intervention; F=Health education; G= Aerobic combined with Resistance exercise; H= Oral nutritional supplementation combined with Aerobic exercise; I= Oral nutritional supplementation combined with Resistance exercise; J= Oral nutritional supplementation combined with Aerobic and Resistance exercise; N= Usual care.

## *3 HB*


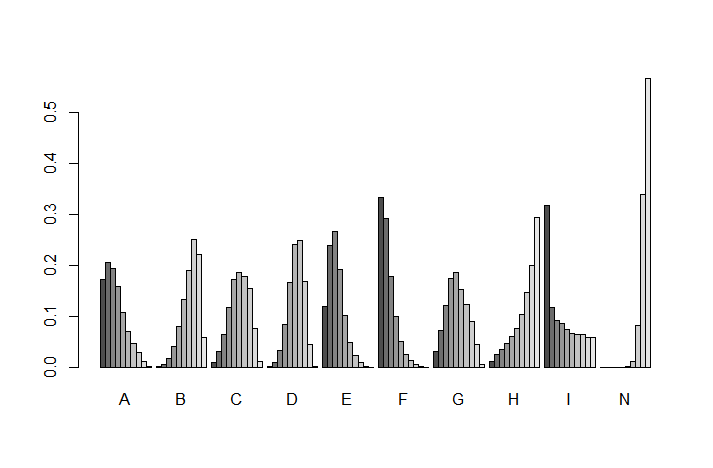

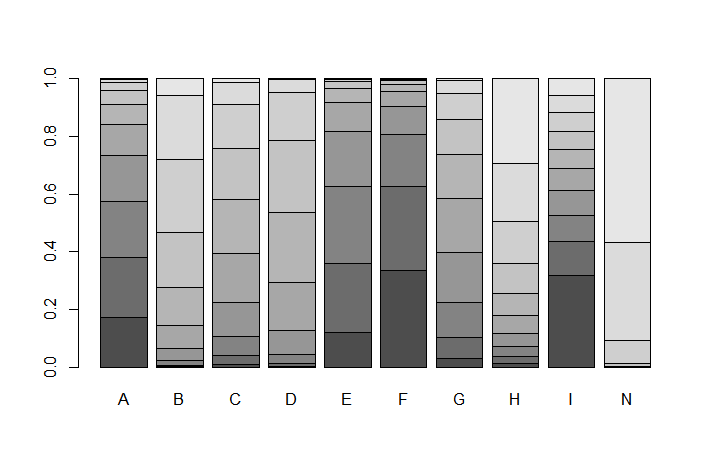


Notes: A= Comprehensive nursing care; B = Resistance exercise; C = Aerobic exercise; D= Oral nutritional supplementation; E = Dietary intervention; F=Health education; G= Aerobic combined with Resistance exercise; H= Oral nutritional supplementation combined with Aerobic exercise; I= Oral nutritional supplementation combined with Resistance exercise; N= Usual care.

# ***Appendix I. Comparison-adjusted funnel plot***

## *1 BMI*


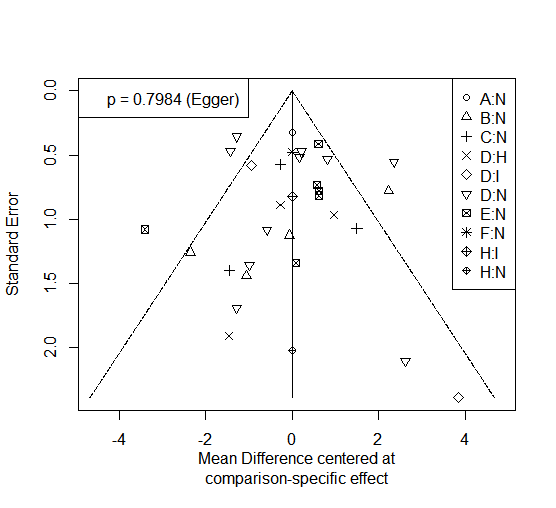


Notes: A= Comprehensive nursing care; B = Resistance exercise; C = Aerobic exercise; D= Oral nutritional supplementation; E = Dietary intervention; F=Health education; H= Oral nutritional supplementation combined with Aerobic exercise; I= Oral nutritional supplementation combined with Resistance exercise; N= Usual care.

## *2 ALB*


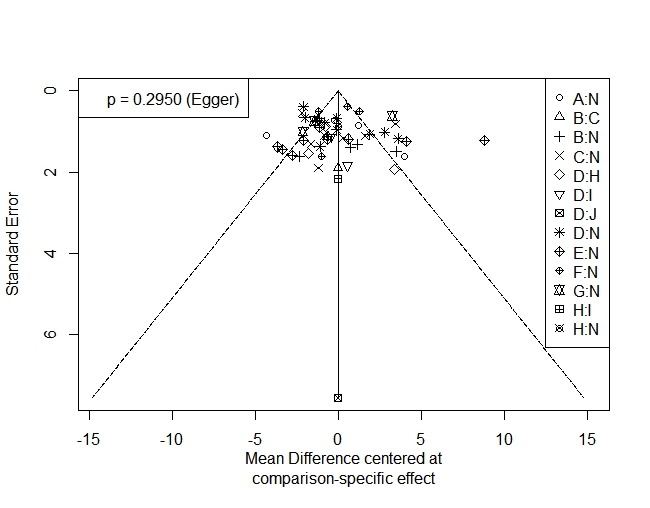
 Notes: A= Comprehensive nursing care; B = Resistance exercise; C = Aerobic exercise; D= Oral nutritional supplementation; E = Dietary intervention; F=Health education; G= Aerobic combined with Resistance exercise; H= Oral nutritional supplementation combined with Aerobic exercise; I= Oral nutritional supplementation combined with Resistance exercise; J= Oral nutritional supplementation combined with Aerobic and Resistance exercise; N= Usual care.

*3 HB*


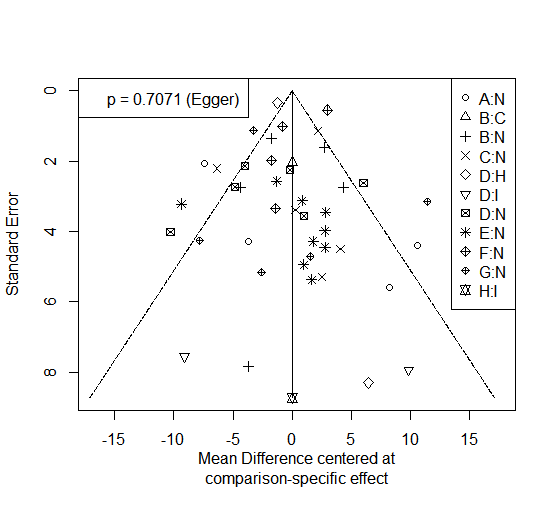


Notes: A= Comprehensive nursing care; B = Resistance exercise; C = Aerobic exercise; D= Oral nutritional supplementation; E = Dietary intervention; F=Health education; G= Aerobic combined with Resistance exercise; H= Oral nutritional supplementation combined with Aerobic exercise; I= Oral nutritional supplementation combined with Resistance exercise; N= Usual care.

# ***Appendix J. Sensitivity Analyses for Outcomes (Results after excluding studies with higher heterogeneity one by one, but excluding individual studies)***

## *1 BMI*

| Format Comparison | | No. | MD (95% CI) | I^2^ statistics |
| --- | --- | --- | --- | --- |
|  | RE vs. UC | 3 | -0.31 [-1.73, 1.10] | 0% |
|  | ONS vs UC | 7 | -0.32 [-1.02, 0.37] | 38% |
|  | DI vs. UC | 5 | 2.40 [1.82, 2.97] | 0% |

## *2 ALB*

| Format Comparison | | No. | MD (95% CI) | I^2^ statistics |
| --- | --- | --- | --- | --- |
|  | CNC vs UC | 2 | 6.43 [5.05, 7.80] | 36% |
|  | RE vs. UC | 5 | 1.79 [0.61, 2.97] | 12% |
|  | AE vs UC | 5 | 1.03 [-0.16, 2.22] | 15% |
|  | ONS vs UC | 6 | 1.15 [0.51, 1.80] | 3% |
|  | DI vs. UC | 8 | 4.55 [3.57, 5.54] | 27% |
|  | HE vs. UC | 3 | 4.47 [3.73, 5.21] | 24% |
|  | AE+RE vs. UC | 2 | 0.04 [-1.12, 1.20] | 0% |

## *3 HB*

| Format Comparison | | No. | MD (95% CI) | I^2^ statistics |
| --- | --- | --- | --- | --- |
|  | CNC vs UC（Excluded J, L and MY, L） | 2 | 18.46 [11.68, 25.25] | 0% |
|  | CNC vs UC（Excluded R L, Z and QM, W） | 2 | 2.10 [-1.53, 5.73] | 0% |
|  | RE vs. UC（Excluded Dai S and R. Afshar） | 3 | 5.65 [2.99, 8.32] | 0% |
|  | RE vs. UC（Excluded Yan X and Cai G） | 3 | 0.44 [-1.92, 2.80] | 0% |
|  | AE vs UC | 4 | 7.48 [5.48, 9.49] | 0% |
|  | ONS vs. UC（Excluded Bolasco et al. 2011, Wen et al. 2022, Y, W et al. 2023） | 4 | 2.65 [0.07, 5.22] | 7% |
|  | ONS vs UC （Excluded Bolasco et al 2011, Wen et al 2022, Y,W et al 2023, R,C et al 2019） | 3 | 1.65 [-1.33, 4.63] | 0% |
|  | HE vs. UC | 3 | 13.70 [12.65, 14.74] | 40% |
|  | AE+RE vs. UC | 5 | 6.59 [-0.08, 13.26] | 80% |
|  | AE+RE vs. UC（Excluded M. A. Tabibi） | 4 | 3.31 [1.28, 5.34] | 0% |

Notes: CNC = Comprehensive nursing care; RE = Resistance exercise; AE = Aerobic exercise; ONS= Oral nutritional supplementation; DI = Dietary intervention; HE=Health education; ONS+RE= Oral nutritional supplementation combined with Resistance exercise; AE+RE= Aerobic combined with Resistance exercise; UC = Usual care.

# ***Appendix K.*** ***GRADE Ratings for All Contrasts***

*1 BMI* (*GRADE* *evidence ratings of improvement in BMI for Nonpharmacologic Interventions in MHD patients)*

| Format Comparison | Direct comparison | | Indirect comparison | | Network | |
| --- | --- | --- | --- | --- | --- | --- |
|  | MD（95%CrI) | Quality of evidence | MD（95%CrI) | Quality of evidence | MD（95%CrI) | Quality of evidence |
| CNC VS UC | -0.16 (-2.9,2.6) | low | NA | NA | -0.16 (-2.9,2.6) | low |
| RE VS UC | 0.75 (-1.0,2.4) | very low | NA | NA | 0.75 (-1.0,2.4) | very low |
| AE VS UC | 0.49 (-1.4, 2.4) | low | NA | NA | 0.49 (-1.4, 2.4) | low |
| ONS VS UC | 0.50 (-0.49, 1.5) | very low | NA | NA | 0.50 (-0.49, 1.5) | very low |
| DI VS UC | 1.8 (0.49, 3.1) | low | NA | NA | 1.8 (0.49, 3.1) | low |
| HE VS UC | 1.5 (-1.4, 4.3) | low | NA | NA | 1.5 (-1.4, 4.3) | low |
| ONS+AE VS ONS | 0.67 (-1.3.2.6) | low | NA | NA | 0.67 (-1.3.2.6) | low |
| ONS+RE VS ONS | 1.3 (-1.2.3.6) | low | NA | NA | 1.3 (-1.2.3.6) | low |
| ONS+AE VS UC | -1.6 (-6.4, 3.2) | low | -0.88 (-3.4, 1.7) | low | -1.2(-3.3,1.0) | low |
| ONS+AE VS ONS+RE | 1.1 (-2.0, 4.2) | low | -3.1(-9.1, 2.9) | low | 0.66(-2.1, 3.2) | low |

Notes: CNC = Comprehensive nursing care; RE = Resistance exercise; AE = Aerobic exercise; ONS = Oral nutritional supplementation; DI = Dietary intervention; HE=Health education; ONS+AE= Oral nutritional supplementation combined with Aerobic exercise; ONS+RE= Oral nutritional supplementation combined with Resistance exercise; UC = Usual care; NA = Not Applicated.

*2 ALB* (*GRADE* *evidence ratings of improvement in ALB for Nonpharmacologic Interventions in MHD patients)*

| Format Comparison | Direct comparison | | Indirect comparison | | Network | |
| --- | --- | --- | --- | --- | --- | --- |
|  | MD（95%CrI) | Quality of evidence | MD（95%CrI) | Quality of evidence | MD（95%CrI) | Quality of evidence |
| CNS VS UC | 6.0 (3.4, 8.7) | very low | NA | NA | 6.0 (3.4, 8.7) | very low |
| RE VS UC | 2.1 (0.20, 4.6) | low | NA | NA | 2.1 (0.20,4.6) | low |
| AE VS UC | 1.3 (-0.7.0, 3.3) | very low | NA | NA | 1.3 (-0.7.0, 3.3) | very low |
| ONS VS UC | 2.1 (0.51, 3.8) | very low | NA | NA | 2.1 (0.51, 3.8) | very low |
| DI VS UC | 6.0 (4.3, 7.8) | very low | NA | NA | 6.0 (4.3, 7.8) | very low |
| HE VS UC | 3.7 (1.1, 6.3) | very low | NA | NA | 3.7 (1.1, 6.3) | very low |
| AE+RE VS UC | 1.7 (-1.2, 4.7) | low | NA | NA | 1.7 (-1.2, 4.7) | low |
| ONS+AE VS ONS | -2.0 (-5.1, 1.0) | very low | NA | NA | -2.0 (-5.1, 1.0) | very low |
| ONS+RE VS ONS | 0.13 (-3.7, 4) | moderate | NA | NA | 0.13 (-3.7, 4) | moderate |
| ONS+AE+RE VS ONS | 3.3 (-12, 19) | low | NA | NA | 3.3 (-12, 19) | low |
| RE VS AE | 0.0046 (-6.2, 6.3) | low | -1.4 (-4.8, 1.8) | very low | -1.1 (-3.9, 1.7) | low |
| ONS+AE VS UC | 0.72 (-4.5, 5.9) | low | -0.14 (-4.6, 4.4) | low | -0.073 (-3.4, 3.2) | low |
| ONS+AE VS ONS+RE | 4.0 (-2.5, 11) | moderate | 1.0(-5.5, 7.5), | very low | 2.2(-2.3, 6.7) | moderate |

Notes: CNC = Comprehensive nursing care; RE = Resistance exercise; AE = Aerobic exercise; ONS = Oral nutritional supplementation; DI = Dietary intervention; HE=Health education; ONS+AE= Oral nutritional supplementation combined with Aerobic exercise; ONS+RE= Oral nutritional supplementation combined with Resistance exercise; ONS+AE+RE= Oral nutritional supplementation combined with Aerobic and Resistance exercise; UC = Usual care; NA = Not Applicated.

*3 HB* (*GRADE evidence ratings of improvement in HB for Nonpharmacologic Interventions in MHD patients)*

| Format Comparison | Direct comparison | | Indirect comparison | | Network | |
| --- | --- | --- | --- | --- | --- | --- |
|  | MD（95%CrI) | Quality of evidence | MD（95%CrI) | Quality of evidence | MD（95%CrI) | Quality of evidence |
| CNC VS UC | 9.1 (3, 16) | very low | NA | NA | 9.1 (3, 16) | very low |
| RE VS UC | 3.4(-1.6, 8.4) | low | NA | NA | 3.4(-1.6, 8.4) | low |
| AE VS UC | 5.4 (0.27, 11) | very low | NA | NA | 5.4 (0.27, 11) | very low |
| ONS VS UC | 5 (0.71, 9.4) | very low | NA | NA | 5 (0.71, 9.4) | very low |
| DI VS UC | 9.2 (5.1, 13) | moderate | NA | NA | 9.2 (5.1, 13) | moderate |
| HE VS UC | 11 (5.4, 16) | moderate | NA | NA | 11 (5.4, 16) | moderate |
| AE+RE VS UC | 6.6 (1.2,12) | very low | NA | NA | 6.6 (1.2,12) | very low |
| ONS+AE VS ONS | -2.8 (-12, 5.5) | low | NA | NA | -2.8 (-12, 5.5) | low |
| ONS+RE VS ONS | 3.9 (-8.3, 16) | low | NA | NA | 3.9 (-8.3, 16) | low |
| RE VS AE | -2 (-13, 8.8) | low | 4.2 (-4.1, 13) | very low | 1.9(-4.8, 8.5) | low |
| ONS+AE VS ONS+RE | 19. (-0.57, 39) | low | -7.8(-29, 13) | low | 6.8(-7.1, 21) | low |

Notes: CNC = Comprehensive nursing care; RE = Resistance exercise; AE = Aerobic exercise; ONS = Oral nutritional supplementation; DI = Dietary intervention; HE=Health education; ONS+AE= Oral nutritional supplementation combined with Aerobic exercise; ONS+RE= Oral nutritional supplementation combined with Resistance exercise; AE+RE= Aerobic combined with Resistance exercise; ONS+AE+RE= Oral nutritional supplementation combined with Aerobic and Resistance exercise; UC = Usual care; NA = Not Applicated.
